# Phylogenetic relationships in the *Niviventer*-*Chiromyscus* complex (Rodentia, Muridae) inferred from molecular data, with description of a new species

**DOI:** 10.3897/zookeys.451.7210

**Published:** 2014-10-03

**Authors:** Alexander E. Balakirev, Alexei V. Abramov, Viatcheslav V. Rozhnov

**Affiliations:** 1 Joint Russian-Vietnamese Tropical Research and Technological Centre, Nguyen Van Huyen, Nghia Do, Cau Giay, Hanoi, Vietnam; 2 A.N. Severtsov’s Institute of Ecology and Evolution, Russian Academy of Sciences, Leninskii pr. 33, Moscow 119071, Russia; 3 Zoological Institute, Russian Academy of Sciences, Universitetskaya nab. 1, Saint Petersburg 199034, Russia

**Keywords:** White-bellied rats, Fea’s tree rat, Southeast Asia, Vietnam, molecular phylogeny, taxonomy, new species

## Abstract

Based on molecular data for mitochondrial (Cyt *b*, COI) and nuclear (IRBP, GHR) genes, and morphological examinations of museum specimens, we examined diversity, species boundaries, and relationships within and between the murine genera *Chiromyscus* and *Niviventer*. Phylogenetic patterns recovered demonstrate that *Niviventer* sensu lato is not monophyletic but instead includes *Chiromyscus chiropus*, the only previously recognized species of *Chiropus*. To maintain the genera *Niviventer* and *Chiropus* as monophyletic lineages, the scope and definition of the genus *Chiromyscus* is revised to include at least three distinct species: *Chiromyscus chiropus* (the type species of *Chiromyscus*), *Chiromyscus langbianis* (previously regarded as a species of *Niviventer*), and a new species, described in this paper under the name *Chiromyscus thomasi* **sp. n.**

## Introduction

The genera *Niviventer* Marshall, 1976 and *Chiromyscus* Thomas, 1925 are members of the *Dacnomys* division of the tribe Rattini (Musser and Carleton 1993, 2005). The composition of the *Dacnomys* division was recently subjected to considerable taxonomic revision based on molecular data (Balakirev et al. 2012a,b, 2013) and now includes five other Indo-Sundaic and Philippine genera: *Dacnomys* Thomas, 1916; *Leopoldamys* Ellerman, 1947; *Saxatilomys* Musser et al., 2005; *Tonkinomys* Musser et al., 2006, and *Anonymomys* Musser, 1981 (in the case of the this last genus, based on morphological supposition, as indicated by Musser and Carleton (2005), given lack of genetic data for this species to date). *Niviventer* is the most speciose genus in the *Dacnomys* division. This division has been placed as a sister group to the *Rattus* division based on combined analysis of mitochondrial and nuclear genes (Lecompte et al. 2008). Both of these groups are included in the large phylogenetic clade of Murinae in Southern Asia corresponding to what has been variably called the “Southern-Asian group” [according to Watts and Baverstock (1995)], the *Rattus* sensu lato group [according to Verneau et al. (1998)], or the “*Rattus* group” [according to Steppan et al. (2005) and Jansa et al. (2006)] by various authors.

Taxonomic composition and preliminary views of relationships within the genus *Niviventer* were first established by Musser (1981) as part of a general revision of *Rattus* Fischer, 1803 sensu lato. Along with *Niviventer*, genera such as *Maxomys* Sody, 1936, *Leopoldamys*, *Lenothrix* Miller, 1903, *Dacnomys*, and *Chiromyscus* were separated from *Rattus* based on features of skull structure, such as the configuration of the lateral walls of the cranium above each pterygoid fossa, the details of the construction of the squamosal roots of the zygomatic arches, the position of the posterior margin of the palatal bridge against the third upper molars, the details of the construction of the mesopterygoid fossa, the proportions of the auditory bullae, and other specific skull structures. Initially, fifteen species were recognized by Musser (1981) within the genus, which has been subdivided into two groups/divisions. The “andersoni group” consisted of *Niviventer andersoni* (Thomas, 1911) and *Niviventer excelsior* (Thomas, 1911), and the “niviventer group” included *Niviventer brahma* (Thomas, 1914), *Niviventer eha* (Wroughton, 1916), *Niviventer langbianis* (Robinson & Kloss, 1922), *Niviventer hinpoon* (Marshall, 1976), and *Niviventer cremoriventer* (Miller, 1900). The taxonomic status of a large series of forms, namely *Niviventer niviventer* (Hodgson, 1836), *Niviventer confucianus* (Milne-Edwards, 1871), *Niviventer tenaster* (Thomas, 1916), *Niviventer fulvescens* (Gray, 1847), *Niviventer coninga* (Swinhoe, 1864), *Niviventer rapit* (Bonhote, 1903), *Niviventer lepturus* (Jentink, 1879) and *Niviventer bukit* (Bonhote, 1903) was unclear, and this group was referred to as the “*niviventer* complex”. Although actual species boundaries and taxonomical affiliation for some taxa and morpha have been debated for a long time, at the moment, the generic structure proposed by Musser (1981) is generally accepted by most recent authors (Nowak 1999, Wang 2003, Pavlinov 2005). As indicated in the most recent summary on the taxonomy of Muroidea (Musser and Carleton 2005), the genus comprises 17 species that are subdivided into the “andersoni” and “niviventer” groups, with three additional species, *Niviventer culturatus* (Thomas, 1916), *Niviventer fraternus* (Robinson & Kloss, 1916) and *Niviventer cameroni* (Chasen, 1940), recognized as distinct within the “niviventer group”. *Niviventer bukit* was not been given a specific status. At the same time, recent investigations using cytochrome *b* (Cyt *b*) gene sequences show that the intrageneric structure within the genus *Niviventer* is much more complex than is currently accepted. Three or four additional monophyletic groups can be separated within the genus (Balakirev and Rozhnov 2010, Balakirev et al. 2012a), namely, the “niviventer group”, the “fulvescens group”, the “langbianis group”, and the “andersoni group”, with the additional possibility of tracing other currently unrecognized groups, given that a number of species, especially in the Sundaic Islands, have yet to be investigated.

The monotypic genus *Chiromyscus* is most likely the closest relative to *Niviventer*. The only representative of this genus, *Chiromyscus chiropus* (Thomas, 1891), was first described as *Mus chiropus* from East Burma. This species is morphologically very similar to the Indochinese taxon *Niviventer langbianis* (Musser 1981, Musser and Carleton 1993, 2005, Musser et al. 2006) and the Sundaic taxon *Niviventer cremoriventer* (Musser 1973) and may be confused with them, but it generally exhibits longer molar rows, higher supraorbital and temporal cranial ridges, a bicolored or mottled tail, a more expansive orange pattern on upperparts, and a nail-like claw on each hallux instead of a small claw. Unfortunately, this species is very rare in museum collections, so until recently little information was available about its natural history and only a few specimens were genetically characterized. In this paper, we investigate the diversity and reveal the genus composition and relationships between *Chiromyscus* and *Niviventer*.

## Materials and methods

Newly collected museum specimens investigated here were obtained in Vietnam during a series of field expeditions of the Joint Russian-Vietnamese Tropical Research and Technological Centre between 2007 and 2013 and deposited at the Zoological Museum of Moscow State University (ZMMU, Moscow, Russia) and at the Zoological Institute of the Russian Academy of Sciences (ZIN, Saint Petersburg, Russia). Most specimens were collected by the authors (BAE, AAV). All animals were identified in the field based on external morphology according to field identification manuals (van Peenen et al. 1969, Musser 1981, Lunde and Nguyen Truong Son 2001, Francis 2008) and the specific traits of skulls that are described in Corbet and Hill (1992) and discussed in Balakirev et al. (2012a). All skulls were investigated later in the laboratory under a stereomicroscope for comparison with more detailed species descriptions (Musser 1973, 1981, Musser and Carleton 1993, 2005). We also studied specimens deposited in the Natural History Museum (BMNH, London, UK), the Museo Civico di Storia Naturale “Giacomo Doria” (MSNG, Genoa, Italy), and the National Museum of Natural History, Smithsonian Institution (USNM, Washington, USA). In total, 32 adult specimens (skulls and/or alcohol-preserved bodies) were examined.

### DNA extraction, PCR amplification, and sequencing

Twenty eight specimens of *Niviventer langbianis* and *Chiromyscus* from 6 localities in Vietnam were sampled for genetic analysis (See Suppl. material 1). Small quantities of liver and muscle tissue or fingertips and earclips were stored in 96% alcohol and used for DNA extraction. Total genomic DNA was extracted using a routine phenol/chloroform/proteinase K protocol (Kocher et al. 1989, Sambrook et al. 1989). The DNA was further purified either by double ethanol precipitation or by using a DNA Purification Kit (Fermentas, Latvia). We targeted four genes that proved to be useful for the phylogenetic analysis of various groups of the superfamily Muroidea generally (e.g., Serizawa et al. 2000, Jansa and Weksler 2004, Buzan et al. 2011) and for Asiatic murids specifically (Michaux et al. 2002, Suzuki et al. 2003, Jansa et al. 2006, Jing et al. 2007, Lecompte et al. 2008, Pages et al. 2010). These genes included a complete or substantial portion of the Cytochrome B gene (Cyt *b*; 950–1143 bp), a portion of the first exon of Interphotoreceptor Retinoid Binding Protein (IRBP; up to 1610 bp), and a portion of exon 10 of the Growth Hormone Receptor (GHR; 815 bp), all of which were amplified for further analysis. We also analyzed the 5’-proximal 680 bp portion of subunit I of the Cytochrome Oxidase gene (COI), which is generally used for species diagnoses and for DNA-barcoding (Hebert et al. 2003). The Cyt *b* was amplified using H15915R, CytbRglu (Kocher et al. 1989, Irwin et al. 1991), and CytbRCb9H (Robins et al. 2007) primers. The COI gene was amplified using the universal conservative primers BatL5310 and R6036R (Kocher et al. 1989, Irwin et al. 1991). The following universal PCR protocol was used to amplify both of the mtDNA fragments: initial denaturation for 1 min 30 sec at 95 °C, denaturation for 30 sec at 95 °C, annealing for 1 min at 52 °C, and elongation for 30 sec at 72 °C, followed by terminal elongation for 2 min at 72 °C. The PCR reaction was performed in a 30–50 ml volume. The final concentration of the PCR mixture in standard Taq PCR buffer with KCl (Fermentas, Latvia), was as follows: dNTPs – 0.2 mM; MgCl_2_ concentration ranges of 2.0 ± 0.25 mM, 10–12 pmol of each primer, 20–50 ng of total DNA temfig and 1 unit of Taq DNA polymerase (Fermentas, Latvia) per tube. The reaction was performed using a Tercik (DNK-Tehnologia, Russia) thermocycler. The IRBP gene (1000–1610 bp in length) was amplified using the IRBP125f, IRBP1435r, IRBP1125r and IRBP1801r primers, according to the method of Stanhope et al. (1992). A nested PCR technique was applied to amplify the GHR gene, in accordance with Jansa et al. (2009). An approximately 1.0-kb portion of exon 10 from the GHR gene was amplified using the primers GHRF1 and GHRendAlt. This polymerase chain reaction product was re-amplified using the nested GHRF1 primer paired with GHR750R and the GHRF50 primer paired with GHRendAlt. The PCR products were purified using a DNA Purification Kit (Fermentas, Latvia).

The resulting double-stranded DNA products were directly sequenced in both directions using the Applied Biosystems 3130 Genetic Analyzer and the ABI PRISM BigDye Terminator Cycle Sequencing Ready Reaction Kit. All obtained sequences were deposited in GenBank (www.ncbi.nlm.nih.gov/genbank) under the accession numbers KF154023–KF154052 and KF154054–KF154085, and certain COI gene sequences were also uploaded into the BOLD database (www.barcodinglife.org project “Indochinese Muridae”, ICMBA).

We also analyzed 122 gene sequences of *Niviventer* (all “langbianis group” species sequences available, as well as some sequences from other species) and *Chiromyscus* that were available in the GenBank and BOLD databases as of 1 May 2013. Out of these 122 sequences, 35 were for Cyt *b*, 27 were for IRBP, 26 were for GHR, and 34 were for COI. The gene sequences from two outgroup species were used to root the phylogenetic tree [*Mus musculus* L., 1758 (V00711, complete mtDNA genome; AB033711, IRBP; NM001048147, GHR) and *Rattus rattus* L., 1758 (EU273707, complete mtDNA genome; AM408328, IRBP; DQ019074, GHR)].

### Sequence editing and phylogenetic analyses

Sequences were aligned using BIOEDIT 3.0 (Hall 1999) and CLUSTAL W (incorporated into BIOEDIT and MEGA 5.05) software and were verified manually. Basic sequence parameter calculations (i.e., variable sites, parsimony-informative sites, base composition biases, nucleotide frequencies and nucleotide substitution tables), codon evolution model testing, and inter- and intra-population divergence evaluations were performed using MEGA 5.05 software (Tamura et al. 2011). All of the most frequently used algorithms, such as maximum parsimony (MP), maximum likelihood (ML), minimum evolution (ME), and neighbor-joining (NJ) were applied to the phylogenetic reconstructions and tree constructions using MEGA 5.05 software. Bayesian inference (BI) was performed using MRBAYES v.3.1 software (Huelsenbeck and Ronquist 2001). The best-fitting models of gene evolution out of 24 possible codon evolution models were determined using a model test module and implemented in MEGA 5.05 using the Maximum Likelihood value (lnL), the Bayesian Information Criterion (BIC) and the corrected Akaike Information Criterion (AICc). The TN93+G+I substitution model was applied for the Cyt *b* and COI genes, and for the combined Cyt *b* + COI data. The GTR+G substitution model was used for the IRBP gene, the GHR gene and for the combined four-gene data set. The calculated gamma shape parameters were 1.82, 1.7084, 0.3979, 0.165 and 0.1697 for the Cyt *b*, COI, IRBP, and GHR genes and for the combined data set, respectively. The robustness of the tree was assessed using a bootstrap procedure with 1000 replications. All of the trees were constructed and visualized directly with MEGA 5.05 or with TREEVIEW 1.6.6 software (Page 1996). We performed the Tajima’s Relative Rate Test (Tajima 1993) to estimate the rate of molecular evolution between species-level branches. No differences between any species-level branches were detected. Intergroup/interspecies genetic divergences (*d*) were calculated under the Tamura 3-parameter (T3P) model using MEGA 5.05 software.

A phylogeny was first estimated for each gene independently, and subsequently for the concatenated dataset once the four genes were manually combined into a single data set in BIOEDIT 3.0 to produce combined samples. This restricted subset (12 variables/taxa in total, see Suppl. material 2) was constructed based on species for which all four genes were available. The TREEROT v.3 program (Sorenson and Franzosa 2007) was used to examine Partitioned Branch Support values (PBS) in order to assess the contribution of each data partition to the combined analysis (Cyt *b*/COI/IRBP/GHR) (Baker and DeSalle 1997). This analysis was performed to test the sustainability of the primary internal nodes in the different genes studied.

Bayesian analysis for the combined data set was performed using four independent runs of 2 × 10^6^ generations each. The most complex substitution model, GTR+G, was used for the combined data set to avoid multi-partition calculation procedures and relax computing process (even though the mitochondrial genes appeared to evolve under the more simple TN93+G+I substitution model). We used a flat Dirichlet prior distribution for the relative nucleotide frequencies and for the relative rate parameters, a discrete uniform prior distribution for the topologies and an exponential distribution for the gamma shape parameter and all branch lengths. A burn-in period of 500,000 generations was determined graphically using TRACER v1.4 (Rambaut and Drummond 2007) to ensure convergence and to ascertain that the runs were not trapped on local optima.

## Results

### Phylogenetic analyses

**Figure 1.**
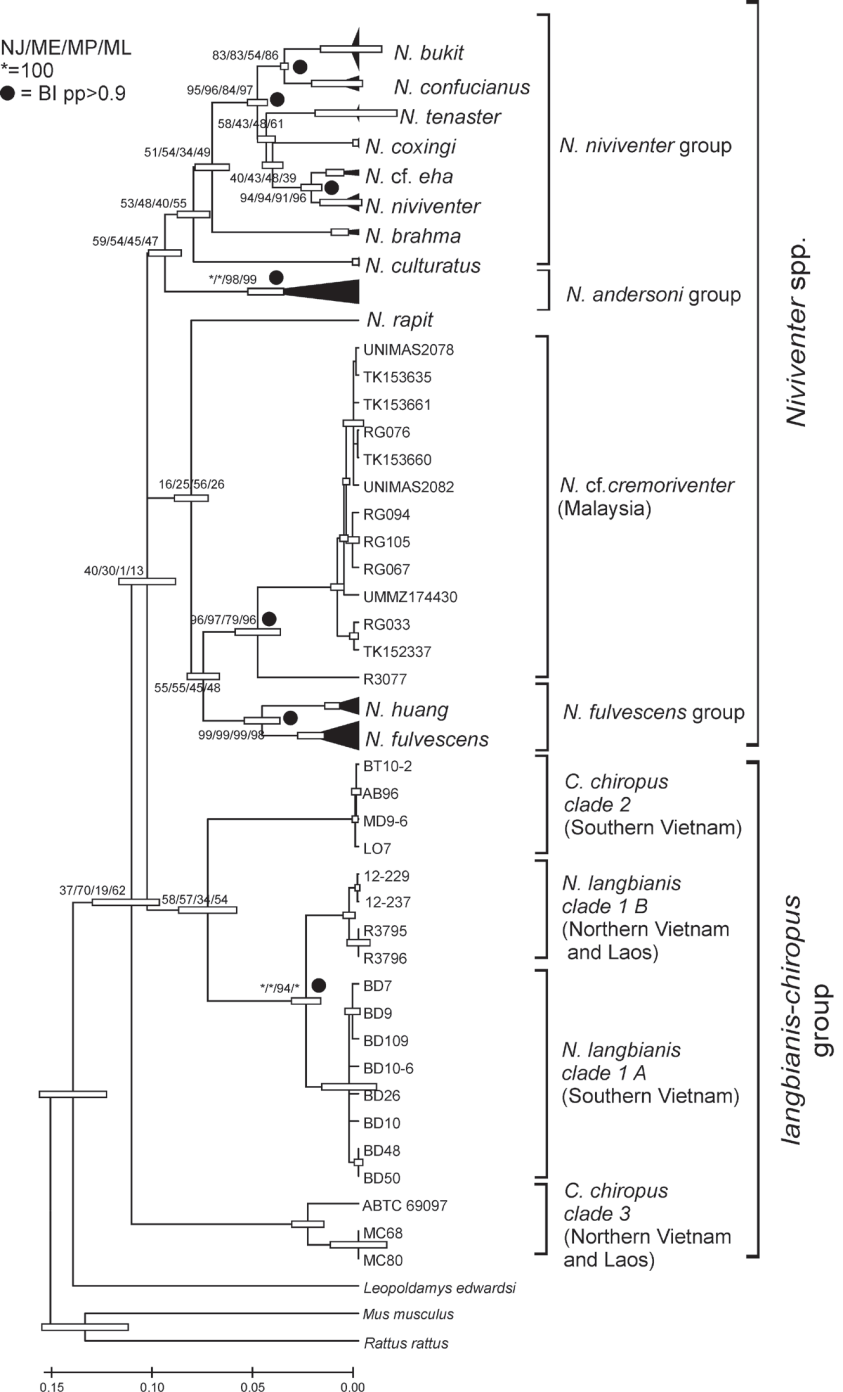
The ultrametric ML phylogenetic tree constructed based on complete Cyt *b* gene sequences (TN93+G+I; 1-2-3 pos. inc.) of the *Niviventer*-*Chiromyscus* complex. The scale bars at the bottom represent the level of divergence (*d*, T3P). The bars at the nodes represent the level of confidence of branch lengths.

Single gene phylogenies revealed that relationships across the overall taxon sampling could not be reliably resolved for most basal nodes, irrespective of the phylogenetic approach (results not shown). The trees obtained from the different genes and methods differed mostly in the topology of the branches of species within the niviventer/fulvescens/langbianis groups of species and in the level of nodal supports. In Fig. 1, we present the ML tree obtained using the Cyt *b* gene and report the support values from ML, ME, NJ, MP and BI analyses. The Cyt *b* phylogeny revealed six multispecies groups in *Niviventer*, as mentioned previously (Balakirev and Rozhnov 2010); namely, “niviventer”, “fulvescens”, “langbianis” and “andersoni” groups, plus two more species level branches, one of which may correspond to *Niviventer rapit* (see Balakirev et al. 2012a, for details), and one for Malayan species which we provisionally refer to here as Niviventer cf. cremoriventer. We previously used the name *Niviventer cremoriventer* (Balakirev and Rozhnov 2010, Balakirev et al. 2012a) for clade 2 within the “langbianis-chiropus” group. However, the samples from mainland Malaysia (named in GenBank as *Niviventer cremoriventer*) constitute an independent specific sister clade to the “fulvescens” group. These samples could not be regarded as conspecific with any of the Vietnamese samples. Due to a lack of comparative morphological materials for Sunda Shelf *Niviventer cremoriventer*, we are unable to discuss the proper attribution and taxonomic position for these samples here. According to Musser (1973), *Niviventer cremoriventer* may represent not a single taxon but a set of vicarious species in the Sundaic region. It is also remarkable that, due to the high level of homoplasy, the “langbianis” group appears to be the most unsustainable group in *Niviventer*. Depending on what species or species-pair was chosen as its representative for comparison with other groups of species in *Niviventer*, and on what phylogenetic method was applied for any single gene and for the combined Cyt *b*+COI data set, the position of the “langbianis” group with respect to other species-groups in *Niviventer* varied considerably. In either case, the bootstrap levels for this branching node were low, preventing any reasonable conclusion about its proper relationships.

**Figure 2.**
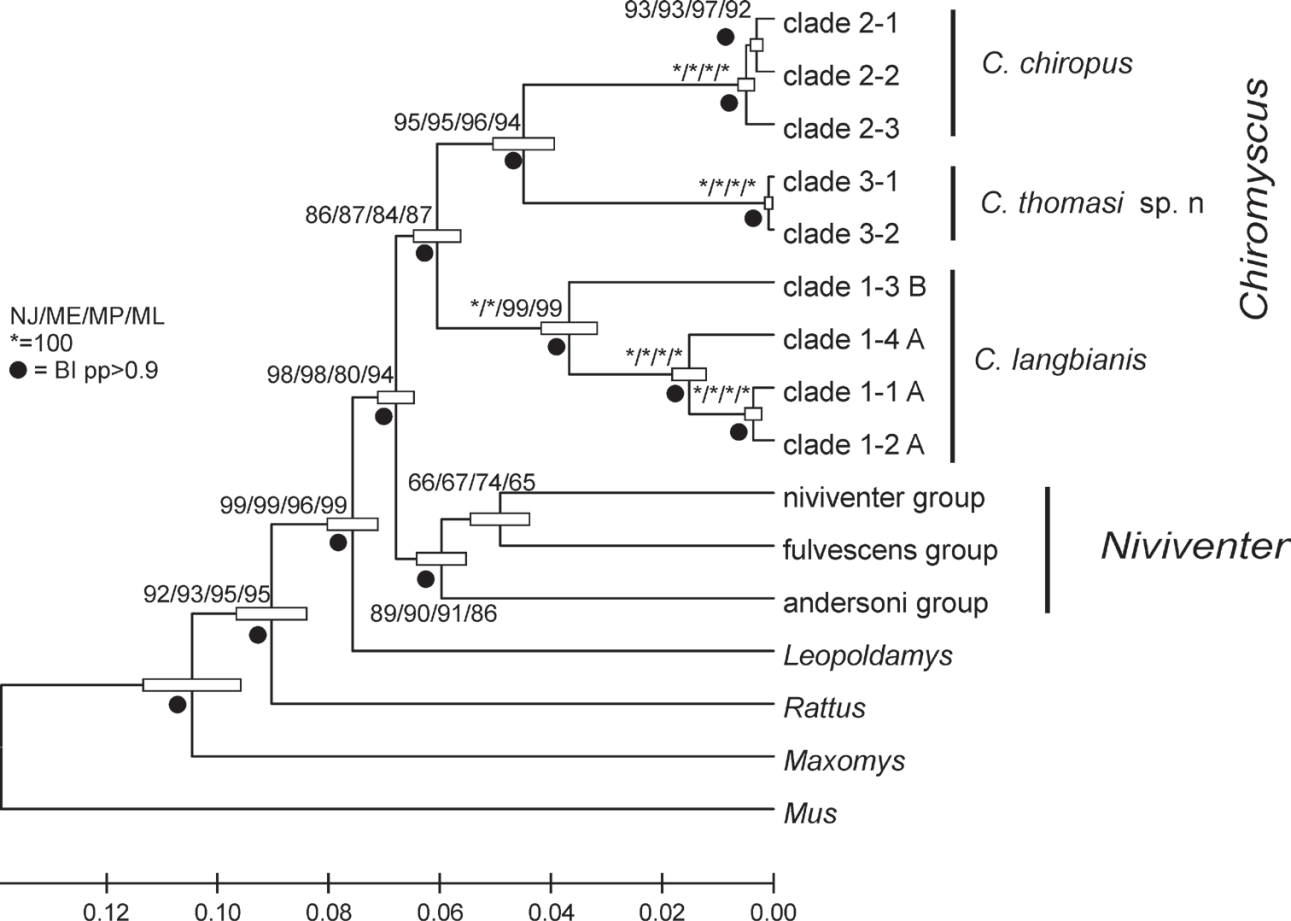
The ultrametric ML phylogenetic tree constructed based on the combined dataset Cytb+COI+IRBP+GHR gene sequences (GTR+G+I; 1-2-3 pos. inc.) of the *Niviventer*-*Chiromyscus* complex. The bars at the nodes represent the level of confidence of branch lengths.

The combined analysis using all four genes on a reduced dataset resulted in a well-supported phylogeny (Fig. 2). Similarly to the individual gene phylogenies, the combined analysis revealed that *Niviventer* is not monophyletic, with *Niviventer langbianis* (clade 1) recovered as sister to *Chiromyscus chiropus* (clades 2) and *Chiromyscus* sp. n. (clade 3). It is also remarkable that both specimens that undoubtedly belong to the genus *Chiromyscus* (Fig. 2) proved to be members of the “langbianis-chiropus” group. This clade appeared to be sister to the genus *Niviventer* and sufficiently genetically divergent to be regarded as a different genus. Thus, the current composition of the genus *Niviventer* did not demonstrate monophyly. Based on the phylogenetic patterns revealed and on the level of genetic subdivisions demonstrated between these three phyla comprising the “langbianis-chiropus” cluster (*d*, T3P = 0.137–0.181 for Cyt *b*; *d*, T3P = 0.074–0.147 for COI), the taxonomic structure and species composition of *Niviventer* and *Chiromyscus* should be revised.

### Taxonomic implications

Given that *Niviventer* is shown not to be monophyletic, two taxonomic approaches to generic nomenclature could be undertaken. Either *Niviventer* could be regarded as a junior synonym of *Chiromyscus* based on taxonomic priority (ICZN 1999, article 23.1) or a revised concept of *Niviventer* could be employed, restricting application of this name to the group of species demonstrating monophyly with the type species of *Niviventer* (*Niviventer niviventer* [Hodgson, 1836]), to the exclusion of those more closely related to the type species of *Chiromyscus* (*Chiromyscus chiropus*), which could be maintained as a separate genus from *Niviventer*. It is a challenge to choose between these two possible decisions. On one hand, *Niviventer* is an established taxonomic name that has been widely and generally used since 1976 for this widespread and taxonomically complex group of rats (up to 17 species), while *Chiromyscus* is arguably a less familiar name in that it has usually been regarded as a rare and monotypic lineage. On the other hand, *Chiromyscus* has priority over *Niviventer*. We consider the principle of stability of nomenclature (ICZN 1999, article 48.1) and the fact that the type species of the genus *Niviventer* falls into the main cluster of species of the “niviventer” group that is paraphyletic with respect to *Chiromyscus*. We also note that the “langbianis-chiropus” species cluster is well differentiated from other *Niviventer* both genetically (*d*, T3P > 0.15 for Cyt *b*; *d*, T3P > 0.08 for COI), and morphologically (see the description below). The close relationship of *langbianis* to *Chiromyscus* was anticipated and earlier postulated by Musser et al. (2006: page 24 and table 3) in their description of *Tonkinomys*. Musser et al. (2006) pointed out that Musser’s 1981 characterization of *Niviventer* was more of a taxonomic summary at the time and meant to be a working hypothesis, not a systematic revision. They also noted that in any future revision of *Niviventer* most species will remain in the genus, but *langbianis* and *chiropus* will likely be separated, and they listed some morphological traits shared by *langbianis* and *chiropus* (including the same number of roots anchoring first upper and lower molars), and noted that both were highly arboreal. Their paragraph closes with this observation: “A revisionary inquiry may either move *Niviventer langbianis* to *Chiromyscus*, or *Chiromyscus chiropus* will be subsumed within *Niviventer* (that name would then be a synonym of the older *Chiromyscus*)”. Based on our field experience, these “langbianis-chiropus” species are indeed primarily arboreal (but may sometimes be trapped on the ground), a characteristic providing an additional ecomorphological basis for the generic identity of *Chiromyscus*. Based on the above considerations, we decided that the latter (restrictive) taxonomical approach would be more reasonable. Therefore, taking into consideration the principle of stability of nomenclature (ICZN 1999, article 48.1) we restrict the content of the genus *Niviventer* sensu stricto to exclude those species most closely related to *Chiromyscus chiropus*. At least three distinct species-level lineages (Fig. 1 and 2) can be allocated to the genus *Chiromyscus*. One of them corresponds to a morphologically easily-distinguished species usually referred to as *Niviventer langbianis* (Musser 1973, Musser and Carleton 1993, 2005, Musser et al. 2006 Balakirev et al. 2012a), whereas the other two include specimens usually attributed to *Chiromyscus chiropus*. However, as seen in Figs 1 and 2, two distinct species can be distinguished among samples of “*Chiromyscus chiropus*” based not only on genetic comparisons but also on the distinguishing morphological features of these animals.

### Morphological analysis and species attribution

Three morphologically distinct groups can be traced from the *Niviventer langbianis*/*Chiromyscus chiropus* complex (i.e., the redefined content of the genus *Chiromyscus*) that correspond to species-level phylogenetic clades revealed within the “langbianis-chiropus” cluster obtained from analyses of mitochondrial and nuclear genes (Figs 1 and 2).

**Figure 3.**
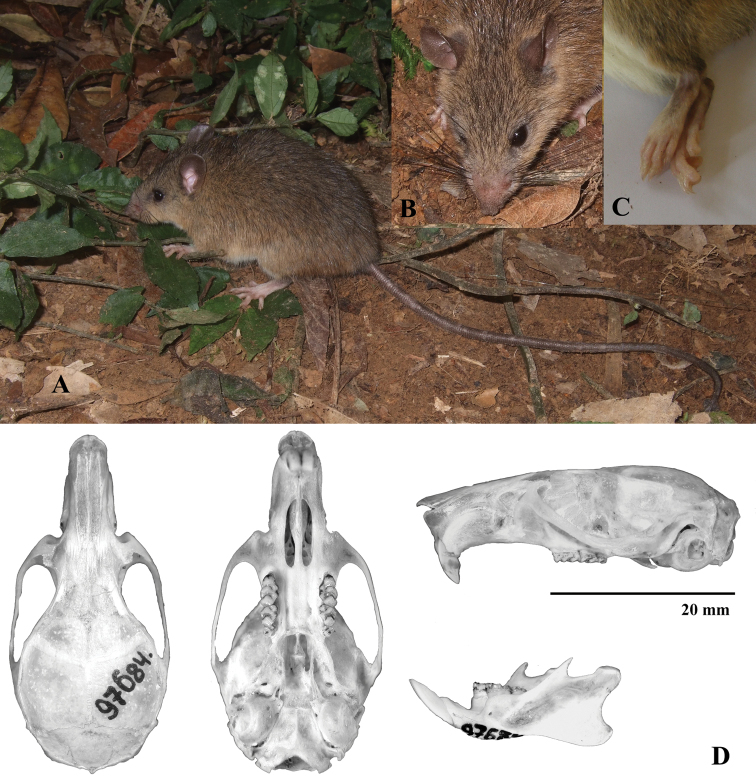
*Chiromyscus langbianis*, Bi Dup-Nui Ba Nature Reserve, Dalat Plateau, southern Vietnam. **A** General appearance (photo by Alexei V. Abramov), ZIN 96679, genetic voucher BD9 **B** Head, dorsal view **C** Hind foot, dorsal view **D** Skull, ZIN 97684.

Thirteen adult specimens identified here as *Chiromyscus langbianis* (Robinson & Kloss, 1922) were collected in the highlands of the Dalat Plateau, Lam Dong Province, southern Vietnam, close to the type locality of this taxon, and in the Huu Lien Nature Reserve, Lang Son Province, northern Vietnam. The corresponding samples formed two independent but closely related clusters, labeled as clade 1 with subclades A and B in Fig. 1. These rats are generally moderate in size (Fig. 3). Their fur is particularly dense, smooth and downy without any spines or guard hairs. The overall color of the dorsal pelage is generally dull and grayish with a touch of fulvous color. The pelage of the belly, as well as of the ventral side of the front legs, is white without any yellowish shade. Occasionally, fulvous or brown spots can be observed. The ventral coloration is sharply separated from the dorsal color. The tail is long and slender and is much longer than the body (135–155% of body length; 140% on average). It is uniformly tinged dark (chocolate) brown from the proximal part to the end. It is well covered with hair, but it lacks a terminal brush. The ears are relatively short, and the vibrissae are particularly long, extending backward well beyond the head. The dorsal sides of the fore and hind feet have a broad brown or chestnut stripe that extends straight beyond the middle part of the foot. The stripe becomes progressively narrower and disappears near the fingers so that the most distal third of the foot and the fingers are completely white. The claws are not so large (about 3.5 mm in length) but are sharp and curved, and adapted for climbing. The hallux bears a nail-like claw. The hallux is not as as perfectly opposable as for the other species of *Chiromyscus*, but is much more mobile in comparison with *Niviventer* species. Generally, *Niviventer langbianis* may be reliably distinguished from the two species discussed below by its dull coloration, its contrasting stripe on the dorsal side of the hind feet and its appreciably narrower and darker tail (Fig. 3A, C). The skull is the most gracile of all species within the genus *Chiromyscus*, and its orbital ridges are not as highly developed as those of other species. Its cranial characters (summarized in Suppl. material 3) have been discussed in detail in Balakirev et al. (2012a). The general appearance of these specimens is consistent with the original description of *Niviventer langbianis* (Robinson & Kloss, 1922). It should be noted that these animals referred to here as *Niviventer langbianis* do not completely correspond to the description of that taxon as presented by Musser (1973, 1981). These animals lack some of the external characteristics, such as an olive hue in the coloration of their upper side or a creamy colored belly. Likewise, according to Musser’s account, the incisive foramina in *Niviventer langbianis* extend to the level of the first molars, but they did not exceed this limit in our specimens from the Dalat Plateau. Nevertheless, a comparative analysis with another *Niviventer* species inhabiting Vietnam (Balakirev and Rozhnov 2010, Balakirev et al. 2012a), namely *Niviventer niviventer*, *Niviventer fulvescens*, *Niviventer huang*, *Niviventer bukit*, *Niviventer confucianus* and *Niviventer tenaster* convinced us that these specimens should be attributed to *Niviventer langbianis*. None of the other Vietnamese species may be attributed to *Niviventer langbianis* based on the complex of specific features listed in original description (Robinson and Kloss 1922) and much more detailed descriptions made by Musser (1973, 1981).

**Figure 4.**
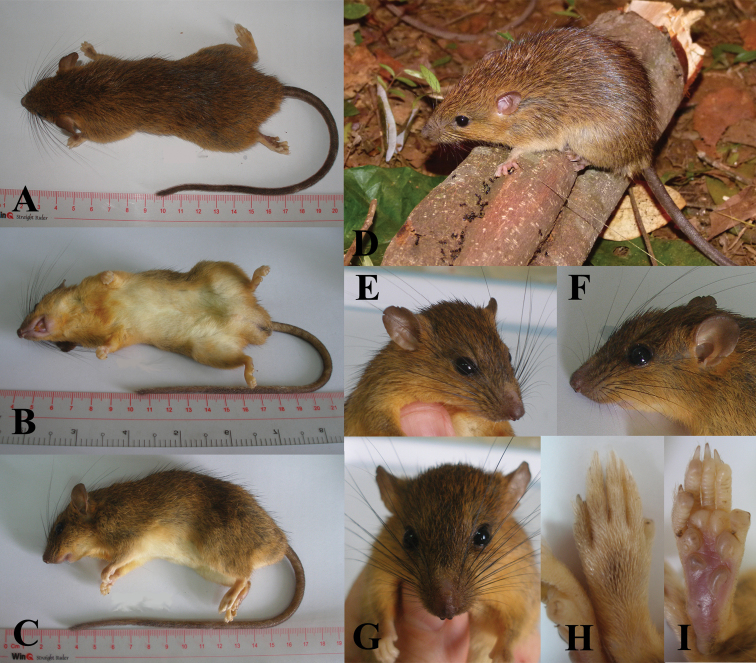
*Chiromyscus chiropus*, southern Vietnam. **A** Dorsal view **B** Ventral view **C** Lateral view **D** General appearance (photo by Alexander E. Balakirev) **E** Head, face-lateral view **F** Head, lateral view **G** Head, dorsal view **H** Hind foot, dorsal view **I** Hind foot, plantar surface. **A–C, E–I** specimen from the Binh Chau Nature Reserve, Ba Ria – Vung Tau Province, southern Vietnam, ZMMU S-191972, genetic voucher BT10-2 **D** specimen from the Bao Loc Forestry, Lam Dong Province, southern Vietnam, ZIN 100966, genetic voucher 12-068.

Another thirteen adult animals we identified as *Chiromyscus chiropus* (Fig. 4) were obtained from various locations in southern Vietnam, specifically, from the Dong Nai National Park (Ma Da Forest), Dong Nai Province; from the Binh Chau Nature Reserve, Ba Ria - Vung Tau Province; from the Lo Go Xa Mat Nature Reserve, Tai Ninh Province and from the Bao Loc Forestry, Lam Dong Province. In our previous publication we attributed these specimens to *Niviventer cremoriventer* (Balakirev et al. 2012a) based on their close morphological similarity with that species and on the fact that these animals do not have the “dark mask” on their face, one of the most obvious morphological features noted as distinctive for *Chiromyscus chiropus* (van Peenen et al. 1969, Corbet and Hill 1992, Lunde and Nguyen Truong Son 2001, Musser and Carleton 1993, 2005, Francis 2008, Lunde 2008). The samples corresponding to this species are labeled as *Chiromyscus chiropus* clade 2 on the phylogenetic tree (Fig. 1). The rats are rather small and brightly colored, clearly distinguishing them from the *Chiromyscus langbianis* (as described above). The coloration of the upper side is a bright fulvous color with a pronounced orange hue, which is most prominent in the humeral area. Their fur is dense, smooth and downy with some blackish flexible guard hairs along a middle line of the back. A prominent buff-orange area separates the dorsal coloration from the creamy-yellowish belly. The sides are more brightly colored than the back. The cheeks, lateral surfaces of the neck and the front legs are a bright yellowish-orange, contrasting with the more dull coloration of the other parts of the body. The dorsal surfaces of both fore- and hindfeet are buffy-orange (Fig. 4H). The finger pads in the fore- and hindfeet are appreciably more developed than in *Chiromyscus langbianis* (Fig. 4I). The claws are larger, and the thumbs of the hind feet bear a plain nail-like claw (Fig. 4H). The tail is long and slender and is much longer than the head-body (128–148% of head-body length; 138% on average). It is uniformly tinged brown from the proximal part to the end and is quite thick and covered with hair. The ears are large and dark-colored; the black vibrissae are long and oriented backward, extending well beyond the ears when laid flat against the head. Their skull morphology (measurements summarized in Suppl. material 3) has been described in detail in Balakirev et al. (2012a).

**Figure 5.**
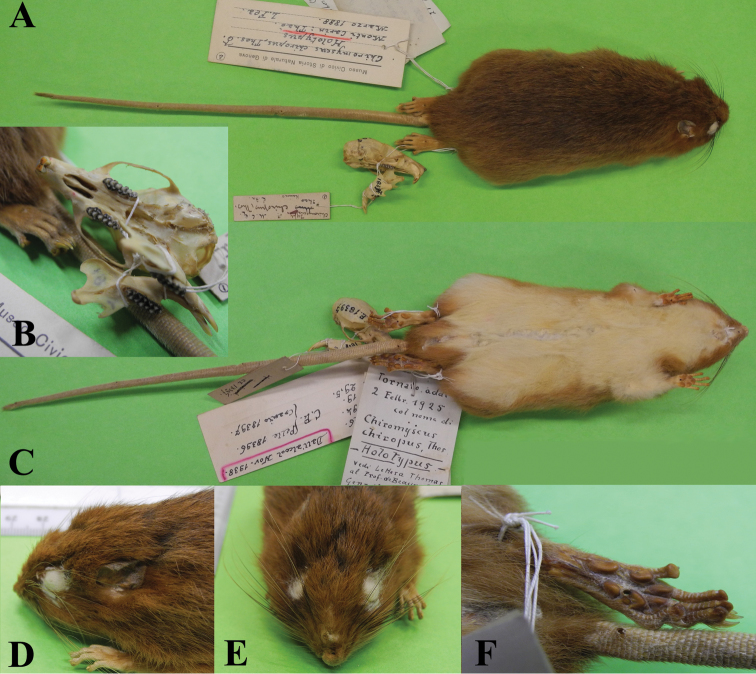
The holotype of *Chiromyscus chiropus*, stuffed skin (MSNG 18396) and skull (MSNG 18397). **A** Stuffed skin, dorsal view **B** Skull, ventral view; hind foot, dorsal view **C** Stuffed skin, ventral view **D** Head, lateral view **E** Head, face view **F** Hind foot, plantar surface. Images were kindly provided by the Museo Civico di Storia Naturale “Giacomo Doria”, Genoa, Italy.

**Figure 6.**
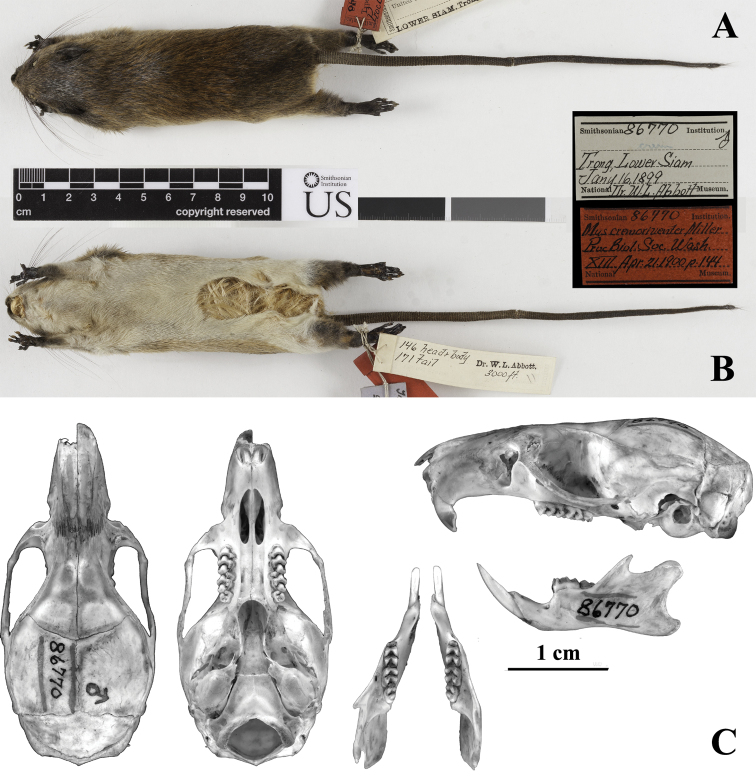
The holotype of *Niviventer cremoriventer*, stuffed skin and skull, USNM 86770. **A** Stuffed skin, dorsal view **B** Stuffed skin, ventral view; the natural coloration of front legs and feet is changed due to chemical treatment **C** Skull. Images were kindly provided by the National Museum of Natural History, Smithsonian Institution, Washington, USA.

*Chiromyscus chiropus* was first described as *Mus chiropus* by Thomas (1891) in East Burma, Carin Hills, Thao (now Myanmar, Karen State, Tao, Karen Hills, also known as the Kayah-Karen Mountains, approximately 80 km NE of Toungoo, near 19°21’N 96°50’E). The holotypes stored in the Museo Civico di Storia Naturale “Giacomo Doria” are MSNG 18396 (skin) and MSNG 18397 (skull). The original description by Thomas (1891) was very general, with only one perceptible diagnostic trait indicated for species recognition, namely, an opposable hallux. The description was as follows: “*Mus chiropus*, sp. n. Similar in size and general appearance to *Mus jerdoni*, Bly., but distinguished from that, as from every other member of the genus by the hallux being opposable as in *Chiropodomys*. Teeth strictly as in *Mus*. Head and body 125; tail 198; hind-foot 30.” A much more explicit description delimiting the genus *Chiromyscus* was made by the same author at a later time (Thomas 1925). The genus was described based on two additional specimens originating from Bao Ha, Tonkin, 300 feet a.s.l. (now Vietnam, Lao Cai Province, Bao Ha, close to 22°10’N, 104°20’E). These two specimens were regarded by Thomas as “unquestionably of the same species” as the Burmese one (Thomas 1925: 504). He wrote as follows: “Now that a well-prepared skin is available, I am able to record that the colour and general appearance are much more striking then was evident on the typical spirit-specimen. For not only is the dorsal colour a warm lined buffy, with the ochraceous lateral band originally mentioned, but the whole side of the cheeks is bright ochraceous, the ochraceous area passing up beyond and behind the ears, forming a bright-coloured patch almost unique in Muridae, and reminiscent of some of the species of *Dremomys*. A rather darker ring around the orbit. Ears short-haired, flesh coloured. The rump, hips, and base of tail are also, like the ear-patches, rich ochraceous”. Unfortunately, no body measurements are listed in the paper. Nevertheless, it should be stressed that the holotype of *Chiromyscus chiropus* (Fig. 6) is obviously outside the size limits (it is substantially smaller) when compared with the rats usually associated with the name *Chiromyscus* (van Peenen et al. 1969, Corbet and Hill 1992, Musser and Carleton 1993, 2005, Lunde and Nguyen Truong Son 2001, Francis 2008, Lunde 2008). It is remarkable that the most prominent features of *Chiromyscus*, a black “mask” or circumorbital “dark rings”, were not mentioned in the original description of *Chiromyscus chiropus* (see Thomas 1891). Thus, despite Thomas’ assertion, it is rather doubtful that Fea’s tree rat from Eastern Myanmar (*Chiromyscus chiropus* proper) and the “mask-bearing” Vietnamese samples are actually members of the same species. We studied images of the skin and skull of the holotype of *chiropus* (kindly provided by the Museo Civico di Storia Naturale). The specimen appeared to be in very good condition, with no perceptible traces of discoloration (Fig. 5). However, the most surprising finding was that two of the most prominent features usually attributed to *Chiromyscus chiropus* (Musser 1981, Corbet and Hill 1992, Francis 2008, Lunde 2008) do not characterize this specimen (Fig. 5D–E). Neither a dark “mask” around the eyes, nor a bicolored tail could be observed. Both the head and tail are unicolored (Fig. 5A–B). The holotype of *Chiromyscus chiropus* is morphologically similar in general appearance, skull and teeth characteristics with our specimens from southern Vietnam, which were previously identified as Indochinese populations of *Niviventer cremoriventer* (Balakirev and Rozhnov 2010, Balakirev et al. 2012a). After analysis of the original description of *Niviventer cremoriventer* description (Miller 1900) and investigation of the holotype (USNM 86770, images kindly provided by USNM; Fig. 6) we concluded that the Vietnamese specimens are not correctly identified as *Niviventer cremoriventer*, a Sundaic species. The skull of the holotype of *cremoriventer* is appreciably more gracile, with undeveloped supraorbital ridges, and the rostrum is considerably more narrow than in our samples from southern Vietnam. The clearest distinctions are in the construction of the pterygoid area, in the position and the shape of the foramina for cerebral nerves and arteries protruding from these bones. All GenBank samples originating from the Malay Peninsula and identified as *Niviventer cremoriventer* constituted a deeply divergent, well supported branch (Fig. 1 and 2) that was closely related not to *Chiromyscus* but rather to the “fulvescens” group within *Niviventer*. Unfortunately, due to a lack of original samples from the Malayan and Sundaic regions, we cannot be completely certain as to the correct species affiliation of these genetic samples, but we suspect these may represent true *Niviventer cremoriventer*.

Unfortunately, we did not have an opportunity to include the holotype of *Chiromyscus chiropus* in our genetic comparisons. Nevertheless, based on apparent morphological similarity we attributed our southern Vietnamese specimens to *Chiromyscus chiropus* proper. Because of the scarcity of museum specimens and DNA-confirmed records for this species, it is difficult to estimate the true distributional range for this species. However, there are no substantial geographic barriers over the vast area stretching from the lowlands of southern Vietnam through Cambodia and central Thailand and west up to the hilly country of Peninsular Thailand and the eastern regions of Myanmar. Thus, there is every reason to believe the species may be distributed over substantial areas in Thailand and Cambodia, most likely scattered over patches of forested areas.

**Figure 7.**
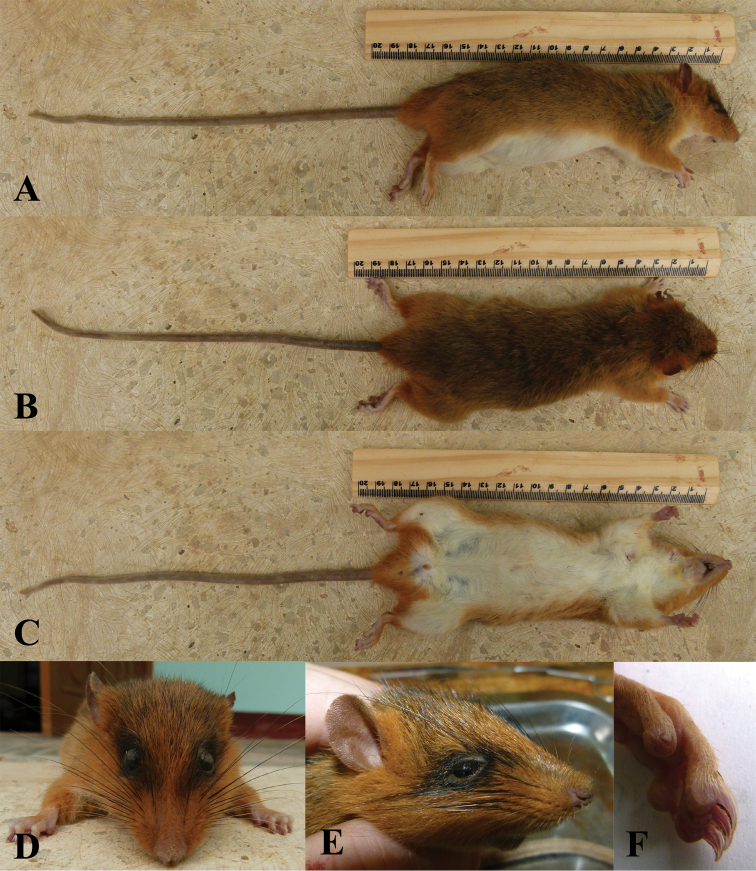
The paratype of *Chiromyscus thomasi* sp. n., Son La Province, northern Vietnam, specimen ZIN 101651, genetic voucher MC80. **A** Lateral view **B** Dorsal view **C** Ventral view **D** Head, face view **E** Head, lateral view **F** Hallux of the hind foot with the nail.

The third distinct species-level genetic lineage within *Chiromyscus* is labeled here as *Chiromyscus chiropus* clade 3 (Fig. 1) and includes two specimens from Son La Province (Northern Vietnam) and one voucher sequence from Northern Laos obtained via GenBank. The general appearance of these Vietnamese specimens is completely consistent with the description of *Chiromyscus chiropus* as detailed in the most recent guides (Francis 2008, Lunde 2008). This is a medium-sized, brightly colored rat (Fig. 7). The coloration of the upper side is a bright fulvous with a perceptible orange hue, which is most prominent in the humeral area. The fur is dense, smooth and downy. Ventrally, the belly, breast and throat are white without any colored patches. The sides are more brightly colored than the back. The cheek, lateral surface of the neck and the front legs are a bright yellowish-orange. A black strip, which is very prominent, passes over the eye, forming a remarkable face “mask” (Fig. 7D, E). The vibrissae are long, both black- and white- colored, and the ears are small (18–20 mm) and rounded. The dorsal sides of both fore- and hindfeet are buffy-orange. The pads of the fore- and hindfeet are as well developed as in *Niviventer cremoriventer*. The claws are large (4.2–5.0 mm in length) and the hallux bears a plain nail instead of a claw. The tail is very long, slender and hairy; it is much longer than the head-body (128–132% of head-body length). It is rather thick, almost uniformly tinged pale-brown from the proximal part to the tip. These specimens are morphologically very similar to specimens from northern Vietnam mentioned by Thomas (1925) in his description of the genus *Chiromyscus*. As discussed above, this taxon is morphologically and genetically different from *Chiromyscus chiropus* and represents a distinct species, which is described below. We can identify no previous taxonomic names applied to this taxon, but three synonyms (*indosinicus*, *vientianensis*, and *quangninhensis*) listed for *Chiromyscus langbianis* in the recent taxonomic summary for mammals (Musser and Carleton 2005) deserve close review and consideration in this context.

Osgood (1932) described *Rattus indosinicus* from northern Vietnam (Sapa, Lao Cai Province). He noted it as being “similar to “*Chiromyscus chiropus* except in smaller size, in less prominent postorbital processes, and in more projecting infraorbital fig…” The coloration of the upper parts is “mixed dusky and ochraceous tawny”, and no “mask” or similar feature is mentioned for this rat. Taking into consideration this description and the skull measurements provided (e.g. greatest length of skull < 38.1 mm for adults) we are convinced the name *indosinicus* is properly attributed to *Chiromyscus langbianis*.

Bourret (1942) described a new rat from the Vientiane region of Laos as *vientianensis*, which [translation from French] “… is more closely related to *Rattus indosinicus* Osgood from Chapa, but has a tail clearly shorter, 99 to 125 per cent the length of the body (average = 111 per cent) instead of 127 to 145 per cent (average = 137 per cent) for the rat of Chapa… An adult male has the hair of the back deep grey at the base and ochre at the extremity… the pelage is fairly coarse; the underside is uniformly cream white, with hairs the same color throughout their length, paws white with a darker median band, not reaching the extremity”. No dark “mask” or bright orange coloration was mentioned by the author. We follow here the opinion of Musser (1973) who ascribed *vientianensis* to the synonymy of *langbianis*.

Dao Van Tien (1970) described *Rattus cremoriventer quangninhensis* from Quang Ninh Province in central Vietnam. Dao Van Tien and Cao Vang Sung (1990) provided a detailed description of this form and made no mention of a face “mask” while noting a shorter tail, less than 125% of head and body length, and flat spines in the pelage. None of these features are characteristic for mask-bearing species of *Chiromyscus*, and we concur with Musser and Carleton (2005) in attributing this nominal taxon to the synonymy of *Chiromyscus langbianis*.

#### Chiromyscus thomasi sp. n.

Taxon classificationAnimaliaRodentiaMuridae

http://zoobank.org/8127C488-5D01-4FFC-9556-0986A1198A26

##### Holotype.

ZMMU S-191982, body in ethanol, skull extracted, genetic code MC68, adult male, collected 17 December 2011 by Alexander E. Balakirev. GenBank IDs: JQ755933, JQ755964, KF154025, KF154068.

**Figure 8.**
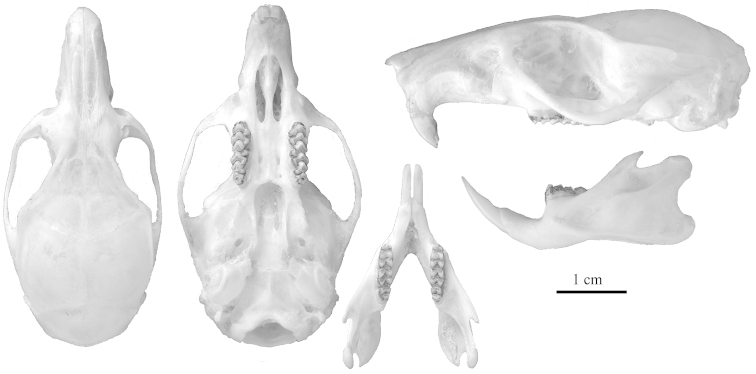
The holotype of *Chiromyscus thomasi* sp. n., Son La Province, northern Vietnam, skull ZMMU S-191982, genetic voucher MC68.

##### Type locality.

Vietnam, Son La Province, Muong Thai Village, near Lung Lo pass, 21°18'31"N, 104°41'34"E, elevation ~ 450 m above sea level.

##### Paratype.

ZIN 101651, body in ethanol, skull extracted, genetic code MC80, adult female, collected 17 December 2011 by Alexander E. Balakirev from the type locality. GenBank IDs: JQ755934, JQ755965, KF154069).

##### Referred material.

BMNH 25.1.1.110, skin and skull, male, Bao Ha, Lao Cai Province, Vietnam; BMNH 26.10.4.167, skin and skull, female, Dak To, Kon Tum Province, Vietnam; BMNH 26.10.4.166, skin, male, Xieng Kuang, Laos.

##### Diagnosis.

This species is set apart from all other described species within the genus *Chiromyscus* by the following combination of morphological traits: (1) Appreciably larger size. This species is the largest in size of any species of *Chiromyscus*. Head and body length is 145–180 mm, tail length 200–231 mm, length of hind foot 27–29 mm, ear length 18–20 mm, greatest skull length 41.0–43.0 mm, upper molar lengths 7.0–8.0 mm; the supraorbital ridges are more developed than in other species, forming a distinct pointed triangle shelf at the point where the frontal and palatal bones come into contact. This shelf is very perceptible in the frontal view of the skull. (2) The upper parts are orange-brown. From the face to behind the ears, the pelage is bright orange, with a prominent darker ring around the eye forming a “mask” on the face. The under parts are pure white and sharply demarcated from the upper parts. The feet and toes are generally white with orange hairs on top. The tail is bicolored, dark on top and appreciably lighter below, where there is a pinkish hue. The hallux is shortened with rounded nails instead of pointed claws. The species is well differentiated genetically from other *Chiromyscus*. The DNA sequences that are deposited in GenBank under IDs JQ755933–JQ755934, JQ755964–JQ755965, KF154025 and KF154068–KF154069 may be used as genetic vouchers for this species.

##### Description.

The fur is dense, smooth and downy. The coloration of the upper side is a bright fulvous with a perceptible orange hue, which is most prominent in the humeral area. On the underside, the belly is pure white without patches or creamy hues. The sides are more brightly colored than the back. The cheek, lateral surface of the neck and the front legs are a bright yellowish-orange. The rump, hips, and base of tail are also, like the cheek, a rich ochraceous color. A very prominent black strip passes over the eye, forming a very characteristic “mask” on the face. The vibrissae are long (over 60 mm), both black- and white-colored, and the ears are small (18–20 mm), pale-brown colored and rounded. The dorsal sides of both the front and hind feet are completely buffy-orange. The pads both in the front and hind feet are well developed. The claws are large (4.2–5.0 mm in length), curved and appreciably sharp. The hallux bears a plain nail instead of a claw. The tail is very long, slender and hairy; it is much longer than the body (128–132% of body length). It is rather thick and almost uniformly tinged pale-brown from the proximal part to the tip.

##### Comparisons.

*Chiromyscus thomasi* is a brightly colored species, a feature that obviously distinguishes it from *Chiromyscus langbianis*, which is generally dull in coloration. With its bright fulvous or orange coloration *Chiromyscus thomasi* is similar to *Chiromyscus chiropus* but may be distinguished from it by its dorso-ventral coloration demarcation line. In *Chiromyscus thomasi*, the white-colored belly replaces the bright orange ventral side coloration abruptly, without any intermediate zone, whereas a lighter-colored fulvous intermediate zone (0.5–1.0 cm in width) is perceptible on the back sides of *Chiromyscus chiropus*. However, the most apparent distinguishing feature of *Chiromyscus thomasi* is a dark “mask” on the face around the eyes, which may be used to visually separate it from any another *Chiromyscus* or *Niviventer* species. *Chiromyscus thomasi* is the largest species in the genus, appreciably bigger than *Chiromyscus chiropus* and *Chiromyscus langbianis*. Its skull well exceeds the known range of size variation for other *Chiromyscus* as well as for the majority of *Niviventer* species, with the exception of *Niviventer tenaster* and the “andersoni” group, both of which are roughly equal in size to, or larger than, *Chiromyscus thomasi*. In comparison with other *Chiromyscus* species, the skull of *Chiromyscus thomasi* is also the most “heavily-built”, with supraorbital ridges that are more developed, forming prominent wide shelves. The skull of *Chiromyscus langbianis* is much smaller and gracile, and the shelves are not so apparent, whereas in *Chiromyscus chiropus* the skull has an obviously convex profile (when viewed from the side), in contrast with *Chiromyscus thomasi*, which appears rather flattened when viewed from the side.

##### Etymology.

The new species is named in honor of Oldfield Thomas (1858–1929), the British zoologist who named and described the genus *Chiromyscus* and the species *chiropus*.

##### Common name.

Thomas' masked tree rat.

##### Distribution.

Confirmed specimens of *Chiromyscus thomasi* have been recorded from the provinces of Son La and Lao Cai in northern Vietnam, the provinces of Kon Tum and Nhge An in central Vietnam, and the provinces of Xieng Khouang and Luang Prabang in northern Laos, based on published data and our (BAE) most recent and unpublished data. This species may have a wider distribution in central Vietnam (Dang Huy Huynh et al. 1994, Dang Ngoc Can et al. 2008) and in northern and central Laos (Aplin et al. 2008, Musser 1981, Corbet and Hill 1992) where similar “mask-bearing” specimens have been reported. It is also likely distributed in south-western China (see Wang 2003) and northern Thailand (see Marshall 1977) but clarifying comparisons are needed to rule out alternative identifications (*Chiromyscus chiropus* and *Chiromyscus langbianis*) before this wider potential geographic distribution is confirmed.

## Conclusion

In spite of the close phylogenetic relationships evident within the *Niviventer*-*Chiromyscus* complex, the taxonomic composition within genera can be reliably resolved by a combination of mitochondrial and nuclear gene analyses, which provide support to the traditional morphological segregation initially suggested for a *langbianis*-*Chiromyscus* cluster by Musser et al. (2006). The patterns revealed show that both *Niviventer* and *Chiromyscus* comprise multiple species and complex phylogenetic composition. Based on the divergence of genetic lineages, we suggest that the genus *Niviventer* sensu stricto can be subdivided into three major sections: the “andersoni” division comprising two species, the “niviventer” division consisting of at least 14 species and the “fulvescens” division comprising two or more species. The identity and position of the Malayan *Niviventer cremoriventer*, which proved to also be related to the “fulvescens” division, remains to be established by additional study (see also Balakirev and Rozhnov 2010, Balakirev et al. 2012a). *Chiromyscus* contains at least three species: *Chiromyscus chiropus* and *Chiromyscus thomasi*, thought of as larger, “mask-bearing species,” and *Chiromyscus langbianis*, which comprises at least two genetic lineages – northern and southern. Taking into consideration the rarity of *Chiromyscus* specimens available and the scarcity of our knowledge about the natural range for the species belonging to this genus, there is reason to believe that the list of species presented remains incomplete.

## Supplementary Material

XML Treatment for Chiromyscus thomasi

## Supplementary materials

Supplementary material 1 Complete list of samples used for phylogenetic reconstructions Data type: species data This dataset is made available under the Open Database License (http://opendatacommons.org/licenses/odbl/1.0/). The Open Database License (ODbL) is a license agreement intended to allow users to freely share, modify, and use this Dataset while maintaining this same freedom for others, provided that the original source and author(s) are credited.Alexander E. Balakirev, Alexei V. Abramov, Viatcheslav V. Rozhnov

Supplementary material 2 The list of samples used for combined *Cyt* b+*COI*+*IRBP*+*GHR* analysis Data type: species data This dataset is made available under the Open Database License (http://opendatacommons.org/licenses/odbl/1.0/). The Open Database License (ODbL) is a license agreement intended to allow users to freely share, modify, and use this Dataset while maintaining this same freedom for others, provided that the original source and author(s) are credited.Alexander E. Balakirev, Alexei V. Abramov, Viatcheslav V. Rozhnov

Supplementary material 3 Cranial measurements, range, and standard deviation Data type: species measurements Explanation note: Cranial measurements, including range and standard deviation (SD), for *Chiromyscus* species from Vietnam (intact, adult skulls of both sexes). This dataset is made available under the Open Database License (http://opendatacommons.org/licenses/odbl/1.0/). The Open Database License (ODbL) is a license agreement intended to allow users to freely share, modify, and use this Dataset while maintaining this same freedom for others, provided that the original source and author(s) are credited.Alexander E. Balakirev, Alexei V. Abramov, Viatcheslav V. Rozhnov
