# Supplementary material for: Phylogenetic relationships in the Niviventer-Chiromyscus complex (Rodentia, Muridae) inferred from molecular data, with description of a new species
Source: Zookeys. 2014 Oct 3;(451):109–36. doi: 10.3897/zookeys.451.7210 (PMC4258623; doi:10.3897/zookeys.451.7210)
Supplement: Supplementary material 1 — Complete list of samples used for phylogenetic reconstructions [file zookeys-451-109-s001.doc]

**APPENDIX 1. The complete list of samples used for phylogenetic comparisons.**

| Genetic sample code | Museum Voucher | Species | Locality | Genes | | | | Source |
| --- | --- | --- | --- | --- | --- | --- | --- | --- |
|  |  |  |  | Cyt *b* | COI | IRBP | GHR |  |
| BD7 | ZIN 97678 | *C. langbianis* (A) | Vietnam, Bi Dup-Nui Ba NR | FJ665434 | KF154023 |  | KF154045 | this paper |
| BD9 | ZIN 96679 | *C. langbianis* (A) | Vietnam, Bi Dup-Nui Ba NR | JN105093 | JN105100 | JN105085 | KF154046 | Balakirev *et al.* 2011 |
| BD10 | ZIN 97680 | *C. langbianis* (A) | Vietnam, Bi Dup-Nui Ba NR | GU457006 |  |  | KF154047 | Balakirev & Rozhnov 2010 |
| BD26 | ZIN 97682 | *C. langbianis* (A) | Vietnam, Bi Dup-Nui Ba NR | FJ665437 | KF154024 | KF372974 | KF154048 | this paper |
| BD48 | ZIN 97683 | *C. langbianis* (A) | Vietnam, Bi Dup-Nui Ba NR | GU457007 |  |  | KF154049 | this paper |
| BD50 | ZIN 97685 | *C. langbianis* (A) | Vietnam, Bi Dup-Nui Ba NR | GU457008 |  |  | KF154050 | this paper |
| BD109 | ZIN 97684 | *C. langbianis* (A) | Vietnam, Bi Dup-Nui Ba NR | GU457009 |  |  |  | this paper |
| BD10-6 | S-191973 | *C. langbianis* (A) | Vietnam, Bi Dup-Nui Ba NR | KF154037 |  |  | KF154051 | this paper |
| R3077 |  | *C. langbianis* (A) | Thailand, Kanchanaburi | HM217402 |  | HM217640 |  | Pages *et al.* 2010 |
| CCP-AO 0004 |  | *C. langbianis* (B) | Cambodia, Kaoh Kong |  | JF445224 |  |  | Eger *et al.* unpubl. |
| R3795 |  | *C. langbianis* (B) | Laos, Khammouane | HM217433 | HM217561 | HM217670 |  | Pages *et al.* 2010 |
| R3796 |  | *C. langbianis* (B) | Laos, Khammouane | HM217434 | HM217562 | HM217671 |  | Pages *et al.* 2010 |
| 12-229 | ZIN 101499 | *C. langbianis* (B) | Vietnam, Huu Lien NR | KF154041 |  |  |  | this paper |
| 12-237 | ZIN 101500 | *C. langbianis* (B) | Vietnam, Huu Lien NR | KF154042 |  |  |  | this paper |
| MC68 | S-191982 | *C. thomasi* | Vietnam, Son La Province | JQ755933 | KF154025 | JQ755964 | KF154068 | this paper |
| MC80 | ZIN 101651 | *C. thomasi* | Vietnam, Son La Province | JQ755934 |  | JQ755965 | KF154069 | this paper |
| ABTC 69097 |  | *C. thomasi* | Laos, Houay Khot Station | EU349739 |  | EU349840 |  | Rowe *et al.* 2008 |
| BM049-03 |  | *C. thomasi* | Laos |  | JF444992 |  |  | Eger *et al.* unpubl. |
| BM072-03 |  | *C. thomasi* | Laos |  | JF444991 |  |  | Eger *et al.* unpubl. |
| MD9-6 | S-186116 | *C. chiropus* | Vietnam, Dong Nai NP | GU827392 | KF154026 |  | KF154070 | Balakirev & Rozhnov 2010 |
| AB96 | S-186543 | *C. chiropus* | Vietnam, Dong Nai NP | KF154039 |  |  | KF154071 | this paper |
| BT10-2 | S-191972 | *C. chiropus* | Vietnam, Binh Chau NR | KF154038 | KF154027 |  |  | this paper |
| Lo7 | S-184818 | *C. chiropus* | Vietnam, Lo Go Xa Mat NR | KF154040 | KF154028 |  |  | this paper |
| 12-042 | ZIN 100962 | *C. chiropus* | Vietnam, Bao Loc Forestry |  | KF154030 |  | KF154073 | this paper |
| 12-057 | ZIN 100963 | *C. chiropus* | Vietnam, Bao Loc Forestry |  | KF154031 |  |  | this paper |
| 12-062 | ZIN 100964 | *C. chiropus* | Vietnam, Bao Loc Forestry |  |  | KF372975 |  | this paper |
| 12-063 | ZIN 100965 | *C. chiropus* | Vietnam, Bao Loc Forestry |  | KF154032 |  |  | this paper |
| 12-068 | ZIN 100966 | *C. chiropus* | Vietnam, Bao Loc Forestry |  |  |  | KF154074 | this paper |
| 12-070 | ZIN 100967 | *C. chiropus* | Vietnam, Bao Loc Forestry |  |  | KF372976 | KF154075 | this paper |
| 12-082 | ZIN 100968 | *C. chiropus* | Vietnam, Bao Loc Forestry |  | KF154033 | KF372977 | KF154076 | this paper |
| 12-083 | ZIN 100969 | *C. chiropus* | Vietnam, Bao Loc Forestry |  | KF154034 |  | KF154077 | this paper |
| DQ019067 |  | *C. chiropus* | no data |  |  |  | DQ019067 | Steppan *et al.* 2005 |
| MVZ:186533 |  | *C.* sp.*** | Vietnam | JQ824369 |  |  |  | Conroy *et al.* 2013 |
| UMMZ174430 |  | *N. cremoriventer* | Indonesia, West Kalimantan | HQ877099 |  |  |  | Gorog *et al.* 2004 |
| ROM:102214 |  | *N. cremoriventer* | Indonesia, Kalimantan Timur |  | JF459850 |  |  | Engstrom *et al.* unpubl. |
| ROM:102215 |  | *N. cremoriventer* | Indonesia, Kalimantan Timur |  | JF459851 |  |  | Engstrom *et al.* unpubl. |
| ROM:102242 |  | *N. cremoriventer* | Indonesia, Kalimantan Timur |  | JF459852 |  |  | Engstrom *et al.* unpubl. |
| ROM:101982 |  | *N. cremoriventer* | Indonesia, Kalimantan Timur |  | JF459853 |  |  | Engstrom *et al.* unpubl. |
| ROM:101983 |  | *N. cremoriventer* | Indonesia, Kalimantan Timur |  | JF459854 |  |  | Engstrom *et al.* unpubl. |
| ROM:101984 |  | *N. cremoriventer* | Indonesia, Kalimantan Timur |  | JF459855 |  |  | Engstrom *et al.* unpubl. |
| ROM:101985 |  | *N. cremoriventer* | Indonesia, Kalimantan Timur |  | JF459856 |  |  | Engstrom *et al.* unpubl. |
| ROM:101986 |  | *N. cremoriventer* | Indonesia, Kalimantan Timur |  | JF459857 |  |  | Engstrom *et al.* unpubl. |
| ROM:102009 |  | *N. cremoriventer* | Indonesia, Kalimantan Timur |  | JF459858 |  |  | Engstrom *et al.* unpubl. |
| ROM:102096 |  | *N. cremoriventer* | Indonesia, Kalimantan Timur |  | JF459859 |  |  | Engstrom *et al.* unpubl. |
| UNIMAS2082 |  | *N. cremoriventer* | Malaysia | JF436979 | JF343490 |  |  | Tamrin *et al.* unpubl. |
| RG033 |  | *N. cremoriventer* | Malaysia | JF436981 | JF343484 |  |  | Tamrin *et al.* unpubl. |
| UNIMAS2078 |  | *N. cremoriventer* | Malaysia | JF436992 |  |  |  | Tamrin *et al.* unpubl. |
| RG067 |  | *N. cremoriventer* | Malaysia | JF436993 | JF343491 |  |  | Tamrin *et al.* unpubl. |
| RG076 |  | *N. cremoriventer* | Malaysia | JF436994 | JF343492 |  |  | Tamrin *et al.* unpubl. |
| RG094 |  | *N. cremoriventer* | Malaysia | JF436998 |  |  |  | Tamrin *et al.* unpubl. |
| TK152337 |  | *N. cremoriventer* | Malaysia | JF437021 |  |  |  | Tamrin *et al.* unpubl. |
| TK153660 |  | *N. cremoriventer* | Malaysia | JF437007 |  |  |  | Tamrin *et al.* unpubl. |
| TK153635 |  | *N. cremoriventer* | Malaysia | JF437008 |  |  |  | Tamrin *et al.* unpubl. |
| TK153661 |  | *N. cremoriventer* | Malaysia | JF437009 |  |  |  | Tamrin *et al.* unpubl. |
| EF053002 |  | *N. andersoni* | China, Yunnan | EF053002 |  |  |  | Jing *et al.* 2007 |
| NC_019617 |  | *N. excelsior* | China, Sichuan, Jiulong | NC019617 | NC019617 |  |  | Chen *et al.* 2012 |
| DQ191511 |  | *N. excelsior* | China, Sichuan |  |  | DQ191511 |  | Jansa *et al.* 2006 |
| USNM574372 |  | *N. excelsior* | China |  |  |  | GQ405386 | Heaney *et al.* 2009 |
| K63 | S-184800 | *N. bukit* | Vietnam, Nam Cat Tien NP | FJ665441 | JQ755857 | JQ755957 |  | Balakirev *&* Rozhnov 2010 |
| BD34 | ZIN 97690 | *N. bukit* | Vietnam, Bi Dup-Nui Ba NR | GU456972 |  |  | KF154052 | Balakirev *et al.* 2012a |
| BD95 | ZIN 97691 | *N. bukit* | Vietnam, Bi Dup-Nui Ba NR | GU456973 |  |  | KF154054 | Balakirev *et al.* 2012a |
| Lo4 | S-184814 | *N. huang* | Vietnam, Lo Go Xa Mat | GU456988 |  |  |  | Balakirev *et al.* 2012a |
| K60 | S-184813 | *N. huang* | Vietnam, Nam Cat Tien NP | FJ665440 |  |  |  | Balakirev *&* Rozhnov 2010 |
| K44 | S-184811 | *N. huang* | Vietnam, Nam Cat Tien NP | FJ665439 |  |  |  | Balakirev *&* Rozhnov 2010 |
| BD78 | ZIN 97686 | *N. tenaster* | Vietnam, Bi Dup-Nui Ba NR |  |  |  | KF154055 | this paper |
| BD86 | ZIN 97687 | *N. tenaster* | Vietnam, Bi Dup-Nui Ba NR | GU457010 |  |  | KF154056 | Balakirev *et al.* 2012a |
| BD87 | ZIN 97687 | *N. tenaster* | Vietnam, Bi Dup-Nui Ba NR | GU457011 |  |  | KF154057 | Balakirev *et al.* 2012a |
| R3459 |  | *N. fulvescens* | Thailand, Loei |  |  | HM217657 |  | Pages *et al.* 2010 |
| R4723 |  | *N. fulvescens* | Thailand, Loei |  |  | HM217702 |  | Pages *et al.* 2010 |
| T-1086 |  | *N. niviventer* | Vietnam, Cao Phong |  |  | AM408323 |  | Michaux *et al.* 2007 |
| AM910969 |  | *N. niviventer* | Vietnam, Cao Phong |  |  |  | AM910969 | Lecompte *et al.* 2008 |
| JN009859 |  | *N. fulvescens* | Thailand |  |  |  | JN009859 | Buzan *et al.* 2011 |
| R3212 |  | *Niviventer* sp. | Thailand, Kanchanaburi |  |  | HM217647 |  | Pages *et al.* 2010 |
| NC_005089 |  | *Mus musculus* | lab. strain "C57BL/6J" |  | NC005089 |  |  | Bayona-Bafaluy *et al*. 2003 |
| Mus musculus |  | *Mus musculus* | lab. strain "C57BL/6J" |  |  |  | NM001048147 | Smith *et al.* 1989 |
| Mus musculus |  | *Mus musculus* | no data |  |  | AB033711 |  | Suzuki *et al.* 2000 |
| J011420 |  | *Mus musculus* | no data | J011420 |  |  |  | No data |
| AB033702 |  | *R. rattus* | Europe | AB033702 |  |  |  | Suzuki *et al.* 2000 |
| SH56 | S-188902 | *R. tanezumi* | Vietnam, Sapa | FR775852 | JN105102 | JN105087 |  | Balakirev & Rozhnov 2012 |
| DQ019074 |  | *R. exulans* | Vietnam |  |  |  | DQ019074 | Steppan *et al.* 2005 |
| MD10-26 | S-189459 | *L. revertens* | Vietnam, Dong Nai NP |  |  |  | KF154085 | this paper |
| K26 | S-186925 | *L. revertens* | Vietnam, Nam Cat Tien NR | JQ755898 | JQ755835 |  |  | Balakirev *et al.* 2012b |
| 12-284 | ZIN 101473 | *L. revertens* | Vietnam, Tam Dao |  |  |  | KF154083 | this paper |
| 12-009 | ZIN 100874 | *L. milleti* | Vietnam, Chu Yang Sin NR | JX173158 | JX173174 | JX173166 |  | Balakirev *et al.* 2013 |
| 12-010 (ZIN 100875) |  | *L. milleti* | Vietnam, Chu Yang Sin NR |  |  | JX173167 |  | Balakirev *et al.* 2013 |
| BD10-12 | not assigned | *Maxomys moi* | Vietnam, Bi Dup-Nui Ba NR |  |  |  | KF154082 | this paper |
| BD5 | ZIN 97675 | *Maxomys moi* | Vietnam, Bi Dup-Nui Ba NR | KF154043 | KF154035 | JQ755954 |  | Balakirev *et al.* 2012b |
| BD 98 | ZIN 97677 | *Maxomys moi* | Vietnam, Bi Dup-Nui Ba NR | KF154044 | KF154036 |  |  | this paper |
| BD47 | ZIN 97676 | *Maxomys moi* | Vietnam, Bi Dup-Nui Ba NR | JN105094 | JN105101 | JN105086 | KF154081 | Balakirev *et al.* 2011 |
| R4223 |  | *Maxomys surifer* | Thailand, Loei | HM217445 | HM217572 | HM217682 | HM217682 | Pages *et al.* 2010 |
| JN009858 |  | *Maxomys surifer* | Thailand |  |  |  | JN009858 | Buzan *et al.* 2011 |

* The sequence as appears to be intermediate between *C. thomasi* and *C. langbianis.* Because of uncertainty concerning this sample origin and locality, we failed to arrive at a final attribution.

** S- ZMMU, Zoological Museum of Moscow State University, Moscow, Russia; ZIN, Zoological Institute of Russian Academy of Sciences, Saint-Petersburg, Russia.
