# Supplementary material for: Phylogenetic relationships in the Niviventer-Chiromyscus complex (Rodentia, Muridae) inferred from molecular data, with description of a new species
Source: Zookeys. 2014 Oct 3;(451):109–36. doi: 10.3897/zookeys.451.7210 (PMC4258623; doi:10.3897/zookeys.451.7210)
Supplement: Supplementary material 2 — The list of samples used for combined Cyt b+COI+IRBP+GHR analysis [file zookeys-451-109-s002.doc]

**APPENDIX 2 The list of samples used for combined *Cytb+COI+IRBP+GHR* analysis.**

| **Combined samples code** | **Taxa** | **Genes** | | | | |
| --- | --- | --- | --- | --- | --- | --- |
|  |  | Cyt *b* | | COI | IRBP | GHR |
|  |  | **nucleotides position** | | | | |
|  |  | 1-1140 | | 1141-1862 | 1863-3519 | 3520-4334 |
| **Main dataset** | | | | | | |
| clade 1-1 A | *C. langbianis* | | JN105093 | JN105100 | JN105085 | KF154046 |
| clade 1-2 A | *C. langbianis* | | FJ665437 | KF154024 | KF372974 | KF154048 |
| clade 1-4 A | *C. langbianis* | | HM217434 | HM217562 | HM217671 | KF154051 |
| clade 1-3 B | *C. langbianis* | | HM217402 | HM217561 | HM217640 | KF154051 |
| clade 2-1 | *C. chiropus* | | GU827392 | KF154026 | KF372975 | KF154070 |
| clade 2-2 | *C. chiropus* | | KF154038 | KF154027 | KF372976 | KF154071 |
| clade 2-3 | *C. chiropus* | | KF154040 | KF154028 | KF372977 | KF154076 |
| clade 3-1 | *C. thomasi* | | JQ755933 | KF154025 | JQ755964 | KF154068 |
| clade 3-2 | *C. thomasi* | | JQ755934 | KF154025 | JQ755965 | KF154069 |
| **Reference groups** | | | | | | |
| andersoni group | genus *Niviventer* | | NC019617 | | DQ191511 | GQ405386 |
| fulvescens group | genus *Niviventer* | | FJ665440 | JQ755857 | JQ755957 | JN009859 |
| niviventer group | genus *Niviventer* | | FJ665441 | JQ755857 | JQ755957 | KF154052 |
| *Leopoldamys* | genus *Leopoldamys* | | JQ755898 | JQ755835 | JX173167 | KF154085 |
| *Maxomys* | genus *Maxomys* | | JN105094 | JN105101 | JN105086 | KF154081 |
| *Rattus* | genus *Rattus* | | AB033702 | FR775817 | JN105087 | DQ019074 |
| *Mus* | genus *Mus* | | J011420 | NC005089 | AB033711 | NM001048147 |
