# Supplementary material for: Phylogenetic relationships in the Niviventer-Chiromyscus complex (Rodentia, Muridae) inferred from molecular data, with description of a new species
Source: Zookeys. 2014 Oct 3;(451):109–36. doi: 10.3897/zookeys.451.7210 (PMC4258623; doi:10.3897/zookeys.451.7210)
Supplement: Supplementary material 3 — Cranial measurements, range, and standard deviation [file zookeys-451-109-s003.doc]

**APPENDIX 3.**

**Cranial measurements, range, and standard deviation (SD) for *Chiromyscus* species from Vietnam (intact, adult skulls of both sexes)**

|  | *C. langbianis*; n = 7 | | | *C. chiropus*; n = 9 | | | *C. thomasi*; n = 2 | | |
| --- | --- | --- | --- | --- | --- | --- | --- | --- | --- |
| mm | Mean | Range | SD | Mean | Range | SD | Mean | Range | SD |
| ONL | 35.62 | 34.97-36.75 | 0.6153 | 39.03 | 36.4-41.34 | 1.588 | 41.44 | 40.18-42.7 | 1.7819 |
| BBC | 15.65 | 15.38-15.89 | 0.1937 | 15.55 | 15.42-15.96 | 0.3419 | 16.38 | 16.3-16.46 | 0.1131 |
| HBC | 10.4 | 10.03-10.87 | 0.5071 | 10.63 | 9.52-10.92 | 0.5013 | 11.61 | 11.46-11.76 | 0.2121 |
| ZB | 16.87 | 16.67-17.30 | 0.3380 | 18.30 | 18.48-19.6 | 1.4920 | 20.63 | 20.22-21.03 | 0.5798 |
| IB | 5.80 | 5.5-6.03 | 0.2185 | 6.40 | 6.0-6.52 | 0.1617 | 6.88 | 6.82-6.94 | 0.0848 |
| LR | 11.52 | 11.15-11.92 | 0.3090 | 12.28 | 10.0-13.64 | 0.3350 | 13.61 | 13.4-13.82 | 0.2970 |
| BR | 5.79 | 5.26-6.03 | 0.2609 | 6.77 | 6.38-7.22 | 0.3340 | 7.49 | 7.12-7.86 | 0.5232 |
| BZP | 3.44 | 3.2-3.59 | 0.1558 | 3.86 | 3.42-4.06 | 0.3971 | 4.08 | 3.92-4.24 | 0.2263 |
| LD | 9.12 | 8.97-9.23 | 0.1154 | 10.14 | 9.5-11.54 | 0.8184 | 11.26 | 11.2-11.32 | 0.0849 |
| LIF | 6.21 | 5.77-6.41 | 0.2439 | 7.59 | 6.62-8.38 | 0.6215 | 7.93 | 7.84-8.02 | 0.1273 |
| BIF | 2.53 | 2.3-2.69 | 0.1219 | 3.00 | 2.8-3.22 | 0.1549 | 3.26 | 3.26 | 0 |
| LBP | 5.60 | 5.26-5.90 | 0.2307 | 6.92 | 6.61-7.46 | 0.2784 | 7.42 | 7.26-7.58 | 0.2263 |
| BBP | 3.37 | 3.07-3.72 | 0.1918 | 3.65 | 3.12-3.86 | 0.2434 | 4.16 | 3.98-4.34 | 0.2546 |
| PPL | 11.96 | 11.66-12.3 | 0.2307 | 13.51 | 12.2-14.38 | 0.7315 | 15.16 | 15.14-15.18 | 0.0283 |
| BMF | 2.44 | 2.18-2.56 | 0.1480 | 3.17 | 2.82-3.48 | 0.2156 | 3.46 | 3.4-3.52 | 0.0849 |
| LB | 5.03 | 4.87-5.13 | 0.1162 | 5.14 | 4.82-5.44 | 0.2042 | 5.23 | 5.14-5.32 | 0.1273 |
| CLM1-3 | 5.71 | 5.38-6.41 | 0.3380 | 6.73 | 5.56-7.3 | 0.5202 | 7.77 | 7.7-7.84 | 0.0990 |
| BM1 | 1.76 | 1.73-1.8 | 0.0504 | 1.87 | 1.58-2.08 | 0.1404 | 2.13 | 2.06-2.2 | 0.0990 |
| CLm1-3 | 5.84 | 5.51-6.02 | 0.1938 | 6.50 | 5.78-6.8 | 0.3087 | 7.15 | 6.94-7.36 | 0.2970 |
| Bm1 | 1.55 | 1.54-1.6 | 0.0242 | 1.69 | 1.5-1.9 | 0.1422 | 1.91 | 1.86-1.96 | 0.0707 |

* Cranial measurements according to Musser & Newcomb (1983) and Musser et al. (2006).

Occipitonasal length, or the greatest length of the skull (ONL), zygomatic breadth (ZB), interorbital breadth (IB), length of rostrum (LR), breadth of rostrum (BR), breadth of braincase (BBC), height of braincase (HBC), breadth of zygomatic plate (BZP), length of diastema (LD), length of incisive foramina (LIF), breadth of incisive foramina (BIF), palatal length (palatal bridge) (LBP), breadth across palate at first molars (BBP), postpalatal length (PPL), breadth of mesopterygoid fossa (BMF), length of bulla (LB), crown length of maxillary molar row (CLM1-3), crown length of mandibular row (CLm1-3), crown breadth of M^1^ (BM1), crown breadth of m_1_ (Bm1).

**List of museum vouchers measured.**

***C. langbianis****:* ZIN 97678-97680, 97682-97685 ***C.chiropus:*** ZMMU S- 191973, ZMMU S-186116, ZMMU S-184818, ZMMU S-186543, ZIN 100961-100962, ZIN 100964-100969 ***C. thomasi***: ZMMU S-191982, ZIN 101651

* Stressed vouchers did not measured and do not included into summary above because of young or broken.
